# Spatial anxiety contributes to the dizziness-related handicap of adults with peripheral vestibular disease

**DOI:** 10.3389/fneur.2024.1365745

**Published:** 2024-04-03

**Authors:** Kathrine Jáuregui-Renaud, Dulce Maria García-Jacuinde, Simón Pedro Bárcenas-Olvera, Michael A. Gresty, Aralia Gutiérrez-Márquez

**Affiliations:** ^1^Unidad de Investigación Médica en Otoneurología, Instituto Mexicano del Seguro Social, Mexico City, Mexico; ^2^Departamento de Audiología y Otoneurología, Hospital General del Centro Médico Nacional “La Raza”, Instituto Mexicano del Seguro Social, Mexico City, Mexico; ^3^Division of Brain Sciences, Imperial College London, Charing Cross Hospital, London, United Kingdom

**Keywords:** spatial anxiety, spatial orientation, stress, vestibular, handicap

## Abstract

In subjects with peripheral vestibular disease and controls, we assessed: 1. The relationship between spatial anxiety and perceived stress, and 2. The combined contribution of spatial anxiety, spatial perspective-taking, and individual cofactors to dizziness-related handicap. 309 adults participated in the study (153 with and 156 without peripheral vestibular disease), including patients with bilateral vestibular deficiency, unilateral deficiency (evolution <3 or ≥3 months), Meniere’s disease, and Benign Paroxysmal Positional Vertigo. Assessments included: general health, personal habits, spatial anxiety (3-domains), perceived stress, spatial perspective-taking, dizziness-related handicap (3-domains), unsteadiness, sleep quality, motion sickness susceptibility, trait anxiety/depression, state anxiety, depersonalization/derealization. After bivariate analyses, analysis of covariance was performed (*p* ≤ 0.05). Spatial anxiety was related to unsteadiness and perceived stress, with an inverse relationship with trait anxiety (ANCoVA, adjusted *R*^2^ = 0.27–0.30, *F* = 17.945–20.086, *p* < 0.00001). Variability on perspective-taking was related to vestibular disease, trait and state anxiety, motion sickness susceptibility, and age (ANCoVA, adjusted *R*^2^ = 0.18, *F* = 5.834, *p* < 0.00001). All domains of spatial anxiety contributed to the Physical domain of dizziness-related handicap, while the Navigation domain contributed to the Functional domain of handicap. Handicap variability was also related to unsteadiness, spatial perspective-taking, quality of sleep, and trait anxiety/depression (ANCoVA, adjusted *R*^2^ = 0.66, *F* = 39.07, *p* < 0.00001). Spatial anxiety is related to perceived stress in adults both with and without vestibular disease, subjects with trait anxiety rated lower on spatial anxiety. State anxiety and acute stress could be helpful for recovery after peripheral vestibular lesion. Spatial anxiety and perspective-taking contribute to the Physical and Functional domains of dizziness-related handicap, possibly because it discourages behavior beneficial to adaptation.

## 1 Introduction

Spatial reasoning is an essential function for activities of daily life, which requires a variety of innate aptitudes, supplemented by learned (malleable) skills (1, 2). Spatial abilities include visualizing, mentally rotating, and transforming spatial information (3). Among these, the ability to imagine viewing a scene from another perspective (perspective-taking) can be impaired by aberrant vestibular stimulation (4); while uncompromised vestibular resources are needed for self-rotation estimates (5), mental body transformation (6), spatial awareness for location, directional heading, and movement through the environment (7). Consistently, vestibular dysfunction is related to impairments in spatial memory (8), visuospatial working memory (9), path integration (10), updating orientation (11), and spatial navigation (12). However, spatial abilities may vary according to multiple factors (13), including anxiety (14). Anxiety may also be provoked by vestibular dysfunction (15–18), as well as motion sickness susceptibility (19), and discordant visuo-vestibular interactions and postural instability may elicit fears associated to specific environments (20).

Anxiety implies responses to increase arousal modulate attentional processes and behavioral inhibition (21). It is related to a variety of factors, such as age (22), sex (23), and quality of sleep (24). Trait anxiety is a predisposition to express constant anxiety (25) and promotes the processing of environmental information preparing the organism for response (26). State anxiety refers to hypervigilance in anticipation of a threat; it can be triggered by acute stress and has a function on overcoming environmental challenges and facilitating memory consolidation (27). In potentially dangerous situations, trait anxiety increases the probability of state anxiety (25).

Spatial anxiety is the domain-specific anxiety that is related to spatial reasoning (28). It denotes the fear and apprehension felt when conducting spatial tasks (29), with negative effects on performance (29, 30). Although, spatial anxiety is related to both experience and performance (31), individual differences are partially explained by genetic and environmental factors, including education (32). In patients with bilateral vestibular hypofunction, spatial anxiety is related to impaired spatial memory and navigation performance (12).

Both vestibular dysfunction and anxiety are related to stress (33, 34). The hypothalamus-pituitary–adrenal axis, the cortico-limbic and the sympathetic systems interact with each other to coordinate the stress response (35). In animal models, unilateral vestibular deafferentation activates the stress axis (36), whereas cortisol administration may improve vestibular compensation (37, 38). In healthy human beings, vestibular caloric stimulation increases cortisol levels (39). Nonetheless, to adapt to challenging situations, stress responses include emotional arousal and altered perceptions (40, 41). Acute stress is a trigger of state anxiety, improving the chance to overcome challenges, facilitating memory of relevant information (27), and inducing focused attention (42). Contrariwise, dysregulation of the stress axis can underlie distorted perceptions, including primary dissociative conditions (43); while symptomatic vestibular disease may provoke dissociative misperceptions (44, 45).

Physiological impairment, anxiety, spatial anxiety, stress could all contribute to dizziness-related handicap. However, studies on their combined contribution to the handicap reported by patients with peripheral vestibular disease are scarce. The twofold purposes of this study were: to assess the relationship between spatial anxiety and perceived stress in adults seeking medical care due to peripheral vestibular disease, and to explore the combined contribution of spatial anxiety, perspective-taking, and individual cofactors to the variability of their dizziness-related handicap. Accordingly, we conducted a correlational study assessing spatial anxiety, perceived stress, perspective-taking, and handicap-related to dizziness in adults with/without peripheral vestibular disease, with the following cofactors: demographics, individual habits (alcohol, tobacco, and sleep), symptoms of unsteadiness, motion sickness susceptibility, and symptoms of common mental disorders (anxiety/depression and depersonalization/derealization).

## 2 Materials and methods

### 2.1 Participants

**Figure 1:**
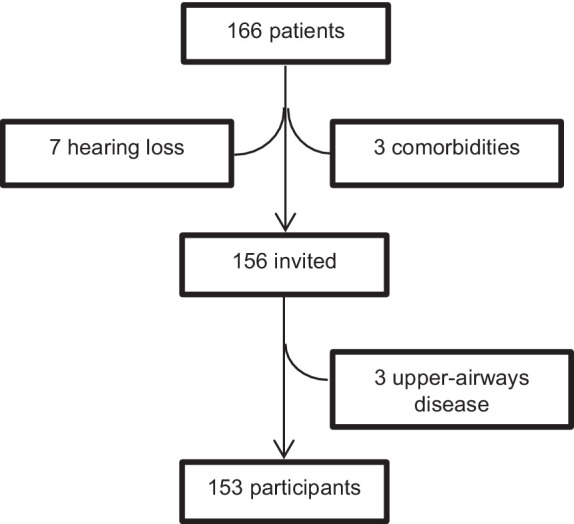
Selection of patients with peripheral vestibular disease.

After approval by the institutional Research and Ethics Committees (IMSS R 2021-3601-219), in a specialized healthcare institutional system (Instituto Mexicano del Seguro Social, Mexico), 309 consecutive participants fulfilling the selection criteria gave their informed consent to participate in the study. They were 153 patients with diagnosed peripheral vestibular disease (18 to 87 years old, 109 women and 44 men) who were referred for specialized evaluation (Figure 1), and 156 volunteers with no history or clinical evidence of vestibular disease (18 to 85 years old, 97 women and 59 men), relatives or companions of patients attending the outpatient clinics. The selection of participants was performed according to the following criteria: no history or medical record of middle ear, retinal, neurological (including migraine), autoimmune or autonomic disorders, or hearing loss >40 dB nHL, or submission to psychiatric care or psychopharmacological treatment and to have completed at least nine years of formal school (secondary school). Three more patients fulfilling the criteria were excluded from the study due to symptoms of acute upper airways disease, at the time of evaluation. The sample size was calculated to assess a correlation value of at least *ρ* = 0.3, with type I error of 0.01 and type II error of 0.1.

The vestibular disease was confirmed at the neuro-otology clinic after at least: clinical assessment (including positional maneuvers), vestibular testing (caloric and/or rotatory tests), and audiological evaluation (middle ear impedance/audiometry/speech audiometry). The general characteristics of the participants are described in Table 1, according to the following groups: participants without history of vestibular disease and participants with diagnosed vestibular disease. Those with vestibular disease were classified by their clinical diagnoses in the following subgroups: bilateral vestibular deficiency, unilateral deficiency with <3 months of clinical evolution, unilateral deficiency with ≥3 months of clinical evolution, Meniere’s disease, and active Benign Paroxysmal Positional Vertigo (BPPV) (before treatment). The frequency of comorbidities in the two groups (without/with vestibular disease) is described in Table 2; compared to participants without vestibular disease, the frequency of corrected refraction errors was less than half in participants with vestibular disease, and no other differences were observed between the groups.

**Table 1:** General characteristics of 156 participants without and 153 with peripheral vestibular disease (by general diagnosis).

	No vestibular disease	Vestibular disease	Unilateral <3 months	Unilateral ≥3 months	Bilateral	Meniere’s	BPPV
Number of participants	156	153	16	42	19	26	49
Men/Women ratio	59/97	44/111	4/12	12/30	4/15	10/16	14/35
Years of age (mean ± S.D.)	54.3 ± 16.4	56.6 ± 15.1	46.7 ± 13.5	53.5 ± 16.3	58.5 ± 12.4	54.9 ± 12.9	63.2 ± 14.3
Body mass index (mean ± S.D.)	27.6 ± 4.3	27.5 ± 4.4	26.4 ± 4.1	27.3 ± 4.1	27.1 ± 3.9	27.8 ± 4.6	28.1 ± 4.8
Years at school (mean ± S.D.)	11.2 ± 2.6	11.3 ± 2.6	11.8 ± 1.6	11.9 ± 2.8	10.5 ± 2.3	11.7 ± 2.9	10.8 ± 2.5
Tobacco smokers (*N*, %)	15 (9%)	8 (5%)	2 (13%)	2 (5%)	2 (10%)	0 (0%)	2 (4%)
Alcohol use (N, %)	28 (18%)	7 (4%)	2 (13%)	2 (5%)	2 (10%)	0 (0%)	2 (1%)
Poor quality of sleep (*N*, %)	76 (49%)	65 (42%)	6 (38%)	17 (40%)	10 (52%)	7 (27%)	24 (49%)

BPPV, Benign Paroxysmal Positional Vertigo; *N*, Number; S.D., Standard Deviation.

**Table 2:** Frequency of comorbidities in the 153 participants with and the 156 participants without peripheral vestibular disease.

Comorbidities	No vestibular disease *N* (%)	Vestibular disease *N* (%)	*p*-value
Corrected refraction errors	53 (44%)	28 (18%)	<0.00001
Diabetes	10 (6%)	16 (10%)	–
High Blood Pressure	29 (18%)	38 (24%)	–
Diabetes & High Blood Pressure	10 (6%)	9 (5%)	–
Thyroid disease	6 (3%)	2 (1%)	–
Thyroid disease & diabetes	1 (1%)	0 (0%)	–
Thyroid disease & High Blood Pressure	0 (0%)	8 (5%)	–
Thyroid disease & diabetes & High Blood Pressure	0 (0%)	1 (1%)	–
Other	14 (8%)	22 (14%)	–

Comparisons were performed using “*t*” test for proportions.

### 2.2 Procedures

After all the participants reported their general health and personal habits (including alcohol and tobacco use) using an in-house questionnaire, the following instruments were administered for self-report:

#### 2.2.1 Pittsburgh sleep quality index

Pittsburgh Sleep Quality Index (46) to assess sleep quality and sleep disturbances. The scale comprises 19 items to generate seven sub-scores on: subjective sleep quality, sleep latency, sleep duration, habitual sleep efficiency, sleep disturbances, use of sleeping medication, and daytime dysfunction. A total score is calculated by the sum of all sub-scores; an overall score of >5 indicates “poor” quality of sleep. The index has shown Cronbach’s alpha coefficient from 0.70 to 0.83 (47).

#### 2.2.2 Motion sickness susceptibility questionnaire

Motion Sickness Susceptibility Questionnaire (short form) (48) to assess individual differences in motion sickness caused by a variety of stimuli (e.g., cars, boats, planes, trains, funfair rides). It contains 18-items, divided into two parts: part A to assess motion sickness during childhood, and part B to assess motion sickness during adulthood. Each sub-score ranges from 0 (no susceptibility) to 27 (maximum susceptibility), and a total score range from 0 to 54, higher scores indicate more susceptibility, with a Cronbach’s alpha coefficient of 0.87 (49).

#### 2.2.3 Unsteadiness rating

Unsteadiness rating. A standardized questionnaire of symptoms related to unsteadiness (50) that includes nine items with no/yes responses. A “no” response is scored 0 points and a “yes” response is scored 1 point, except for vertigo that is scored 2 points. Frequent falls are considered only when ≥1 per month and frequent stumbles are considered only when ≥1 per week. A total score is obtained by summing the ratings for all the items (range 0 to 10). A score ≥4 points has been related to balance disorders (50).

#### 2.2.4 Hospital anxiety and depression scale

Hospital Anxiety and Depression Scale (HADS) (51), which comprises 14 items, 7 for anxiety and 7 for depression, which are rated on a 4-point scale (0 to 3), each score ranges from 0 to 21, and a total score is obtained by summing the ratings for all the items. Cut-off scores of ≥8 (sub-scores) and ≥11 (total score) have shown sensitivities and specificities in the range of 0.70 to 0.90 for anxiety/ depression (51, 52), and Cronbach’s alpha coefficient from 0.67 to 0.93 (52).

#### 2.2.5 State-trait anxiety inventory

The short version of the State-Trait Anxiety Inventory (53) to assess state anxiety, which comprises 6 items coded on a 4-point scale (from 0 to 3). A total score is calculated by the sum of the ratings for all the items (range from 0 to 18), higher scores are related to more anxiety (54).

#### 2.2.6 Depersonalization/Derealization inventory

Depersonalization/Derealization Inventory (55), which comprises 28 items coded on a 5-point scale (from 0 to 4). A total score is obtained by the sum of the individual scores (range from 0 to 112), higher scores are related to more frequency/severity of depersonalization/derealization symptoms, with an internal consistency coefficient of 0.95 (55).

#### 2.2.7 Perceived stress scale-10

Perceived Stress Scale-10 (56), which is a measure of global perceived stress. It contains 10 items that are coded on a 5-point scale (from 0 to 4). A total score ranging from 0 to 40 is computed by reverse scoring the four positively worded items and then summing the ratings for all the items, higher scores are related to more perceived stress, with a Cronbach’s alpha coefficient of 0.78 (57).

#### 2.2.8 Spatial anxiety scale

Spatial Anxiety Scale (31), which includes three subscales assessing anxiety on spatial mental manipulation (8 items), spatial navigation (8 items), and spatial imagery (8 items). It comprises a total of 24 items that are scored on a 4-point scale (from 0 to 4). A total score is obtained by summing the ratings for all the items, higher scores are related to more spatial anxiety, with Cronbach’s alpha coefficient >0.8 (31).

#### 2.2.9 Object perspective test

Object Perspective Test (58) in which participants rely on an egocentric frame of reference and form egocentric representations to solve the task. It is a pencil-and-paper test that comprises 12 items showing an array of objects and an “arrow circle”; participants are asked to imagine themselves facing a particular direction within the array, and then they are questioned about the direction between some of the objects. Absolute errors are calculated by the difference in degrees between the correct answer and what they draw in the “arrow circle.” Then a total score is obtained by the average for all the responses, higher scores indicate larger errors, with a total accuracy score of *r* = 0.77 (59).

#### 2.2.10 Dizziness handicap inventory

Dizziness Handicap Inventory (60) to evaluate the self-perceived handicapping effects by dizziness and unsteadiness. It comprises 25 items that are scored on a 4-point scale (from 0 to 4). A total score is obtained by summing the ratings for all the items that were originally sub-grouped into three content domains, representing physical (7 items), emotional (9 items), and functional (9 items) aspects of handicap (60). Though, recent evidence suggests the need to reassess the factorial structure of the inventory (61, 62).

### 2.3 Experimental design and statistical analyses

A cross-sectional correlational study was designed. Assessment of data distribution was performed using the Kolmogorov Smirnov test. Accordingly, the bivariate analysis was performed using either: Mann Whitney U test or *t*-test (for means or for proportions); Spearman correlation or Pearson’s correlation coefficients; Kruskal Wallis analysis or analysis of variance (ANOVA) with Tukey honest significance test (HSD) for unequal N (Spjotvoll/Stoline test). The multivariate analysis was performed using analysis of covariance (ANCoVA), which was designed to compare participants with bilateral vestibular deficiency versus each of the other subgroups of participants. All the tests were performed using a two tailed significance level of 0.05.

## 3 Results

### 3.1 Bivariate analysis

#### 3.1.1 Analysis by the general characteristics of the participants

Participants with/without vestibular disease had similar age and body mass index (*p* > 0.05) (Table 1). However, within the group of participants with vestibular disease, those with unilateral deficiency (either <3 months or ≥3 months of clinical evolution) were the youngest, while those with BPPV were the eldest (ANOVA, *F* = 5.397, *p* = 0.0004; Tukey HSD test for unequal N, *p* = 0.02). Linear correlation was observed between the age and the scores on: motion sickness susceptibility, perceived stress, quality of sleep and the anxiety sub-score of the HADS (Pearson *r* values from −0.12 to 0.20, *p* < 0.03).

The proportion of women was similar in the two main groups (with/without vestibular disease) and the mean years at formal school was the same for all subgroups (*p* > 0.05). Comparisons by sex on the instrument scores showed that, compared to men, women had higher scores on the symptoms related to unsteadiness (Mann Whitney U test, *Z* = −2.916, *p* = 0.003), the Navigation domain of spatial anxiety (Mann Whitney U test, *Z* − 2.315, *p* = 0.02), the three domains of the dizziness-related handicap instrument (Mann Whitney U test, *Z* from 1.963 to 2.099, *p* < 0.05), the sleep quality index (Mann Whitney U test, *Z* − 3.161, *p* = 0.001), and the sub-scores and total score of the HADS (Mann Whitney U test, *Z* from 1.963 to 2.577, *p* < 0.05). However, when a similar analysis by sex was performed just in the group of participants with vestibular disease, only the increased score on the sleep quality index in women (compared to men) persisted (Mann Whitney U test, *Z* − 2.749, *p* = 0.006), with a borderline result on the HADS depression sub-score (Mann Whitney U test, *Z* − 1.955, *p* = 0.0506).

For participants, the report of tobacco or alcohol use was low, particularly among those with vestibular disease (Table 1); while one third to half of the participants of any group or subgroup reported bad quality of sleep (Sleep Quality Index >5) (Table 1).

#### 3.1.2 Analysis by groups and subgroups

**Figure 2:**
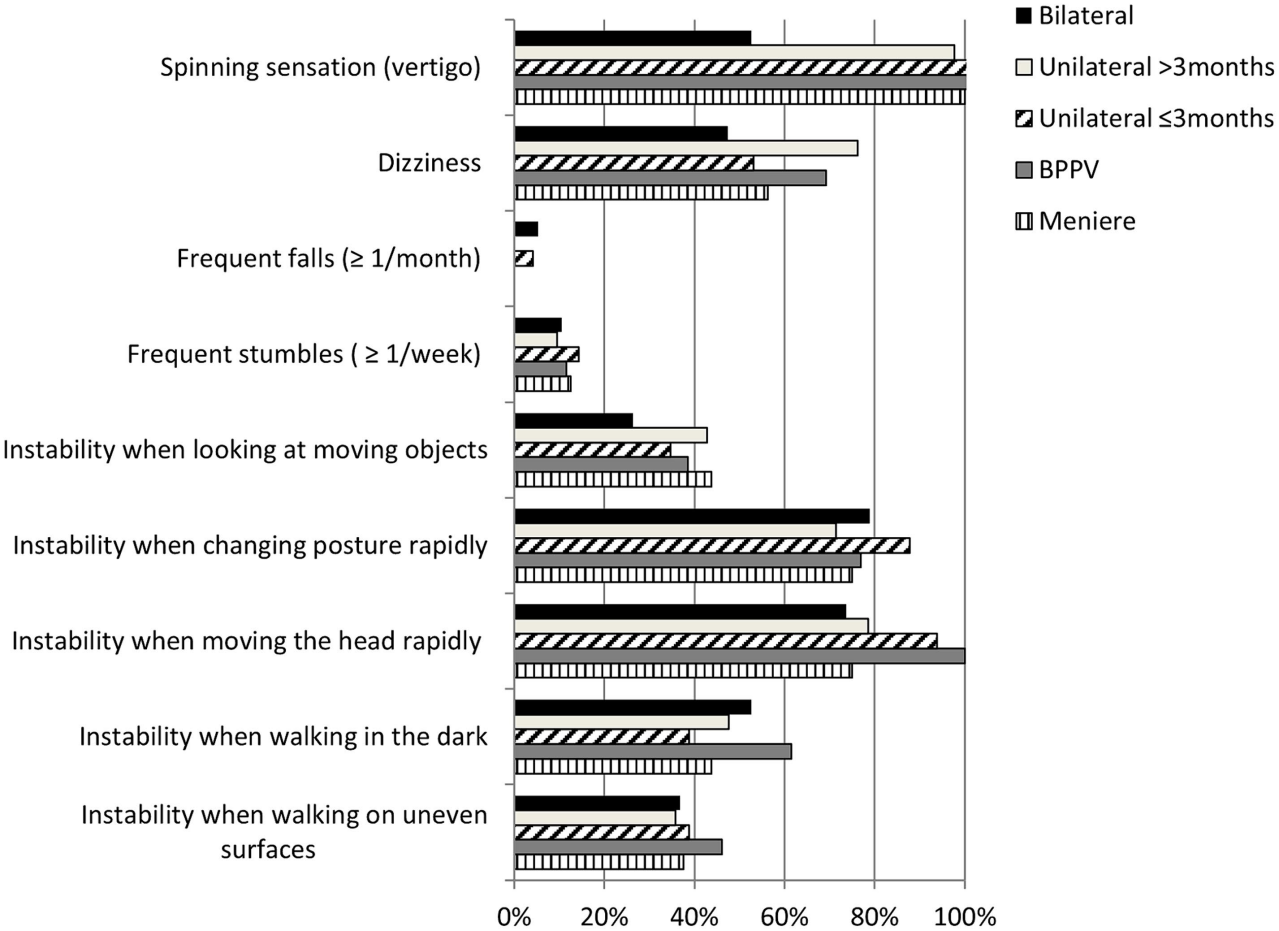
Frequency of symptoms related to unsteadiness of 153 patients by general diagnosis. (BPPV, Benign Paroxysmal Positional Vertigo).

On the questionnaire of symptoms related to unsteadiness, participants without vestibular disease reported almost no symptoms (Table 3) and among the subgroups of participants with vestibular disease the total score was similar (*p* > 0.05) (Table 3). The most frequent symptom was vertigo, except for the group of patients with bilateral vestibular disease, which reported vertigo with half the frequency than the other four subgroups of patients; in contrast, less than one fifth of the patients with vestibular disease of any subgroup reported frequent stumbles or frequent falls (Figure 2). Contrariwise, more than two thirds of the patients with vestibular disease, of any subgroup, reported instability when moving the head rapidly, or when changing posture rapidly (Figure 2).

**Table 3:** Median and Quartiles 1 and 3 (Q1–Q3) of the scores on the instruments administered to 156 participants without and 153 with peripheral vestibular disease (by general diagnosis).

Subgroups	No vestibular	Vestibular	Unilateral <3 months	Unilateral ≥3 months	Bilateral	Meniere’s	BPPV
	(*N* = 156)	(*N* = 153)	(*N* = 16)	(*N* = 42)	(*N* = 19)	(*N* = 26)	(*N* = 49)
Instruments	Median (Q1–3)	Median (Q1–3)	Median (Q1–3)	Median (Q1–3)	Median (Q1–3)	Median (Q1–3)	Median (Q1–3)
Symptoms related to unsteadiness	0 (0–1)	5 (3–6)**	4.5 (2–6)	5 (3–6)	4 (2–4)	5 (4–6)	5 (4–6)
Pittsburgh Sleep Quality Index	5.5 (3–8)	5 (3–7)	4 (2–6)	5 (3–7)	4.5 (3–7)	5 (3–7)	5 (3–11)
Anxiety and Depression							
Anxiety sub-score	2 (0–5)	2 (0–4)	3.5 (1–5)	3 (1–6)	2 (0–4)	1 (0–4)	1 (0–2)
Depression sub-score	0 (0–2)	1 (0–3)*	1 (0–2.5)	1 (0–3)	1 (0–3)	1 (0–4)	1 (0–2)
Total score	3.5 (0–6)	3 (1–7)	4 (1–6.5)	5 (2–8)	3 (0–8)	2 (1–7)	2 (0–4)
State-Trait Anxiety (short version)	3 (2–6)	6 (5–9)**	7.5 (4.5–9.5)	6 (5–8)	6 (3–9)	7 (6–9)	6 (4–8)
Depersonalization/derealization	0 (0–4.5)	2 (0–3)	2 (1–3.5)	2 (1–4)	2 (0–2)	1 (0–2)	1 (0–2)
Perceived Stress	10 (4–15)	10 (6–17)	9 (6–20)	13 (8–19)	7 (2–16)	9.5 (4–13)	9 (4–16)
Motion sickness							
Motion sickness before age 12	1 (0–3)*	0 (0–2)	0 (0–2)	0 (0–3)	0 (0–2)	0 (0–3)	0 (0–2)
Motion sickness last 10 years	0 (0–2)	0 (0–1)	0 (0–0)	0 (0–2)	0 (0–0)	0 (0–0)	0 (0–1)
Motion sickness Total score	1 (0–6)*	0 (0–3)	0 (0–2)	2 (0–4)	0 (0–2)	0 (0–6)	0 (0–2)
Spatial anxiety							
Spatial anxiety Navigation	6 (2–11.5)	16 (8–22)**	17.5 (7.5–23)	18 (10–22)	14 (7–20)	13 (7–21)	15 (10–21)
Spatial anxiety M-manipulation	5 (2–11.5)	15 (9–21)**	18 (9.5–23)	16.5 (10–21)	14 (6–21)	13 (9–22)	14 (7–21)
Spatial anxiety Imagery	5 (2–10)	14 (8–21)**	16 (6–21)	16.5 (9–21)	12 (7–19)	13 (7–20)	15 (8–22)
Spatial Anxiety Total	15.5 (9–31)	46 (24–64)**	51 (20.5–67.5)	50.5 (28–65)	40 (20–60)	37.5 (24–62)	41 (26–63)
Dizziness-related handicap							
Physical Handicap	0 (0–0)	12 (8–16)**	15 (6–23)	10 (6–18)	12 (6–14)	12 (8–14)	14 (8–18)
Functional Handicap	0 (0–0)	8 (4–16)**	18 (9–25)	8 (4–14)	12 (4–18)	4 (2–12)	6 (2–10)
Emotional Handicap	0 (0–0)	10 (6–18)**	17 (7–27)	10 (6–20)	10 (4–16)	8 (4–14)	8 (6–16)
Total Handicap	0 (0–0)	28 (20–48)**	50 (25–74)	28 (18–50)	32 (20–42)	27 (20–36)	26 (20–38)

Higher scores by group comparison are marked with asterisks (*= p<.05, and ** p<.005), according to Mann Whitney U test. (BPPV= Benign Paroxysmal Positional Vertigo; N= Number; S.D. = Standard Deviation).

Comparisons between the two groups (with/without vestibular disease) on the scores on symptoms of common mental disorders showed that the group with vestibular disease had higher scores on: state anxiety (Mann Whitney U test, adjusted *Z* = 7.514, *p* < 0.00001) and depression (HADS sub-score) (Mann Whitney U test, adjusted *Z* = 1.979, *p* = 0.04) (Table 3); with no differences among the subgroups of participants with vestibular disease (*p* > 0.05). For depersonalization/derealization, the score was similar among the groups and subgroups, but it was related to the scores on: anxiety/depression (HADS) (Spearman *r* values from 0.36 to 0.46, *p* < 0.00001), symptoms related to unsteadiness (Spearman *r* = 0.31, *p* < 0.00001), perceived stress (Spearman *r* = 0.31, *p* < 0.00001), motion sickness susceptibility (Spearman *r* values from 0.23 to 0.32, *p* < 0.00001), spatial anxiety (Spearman *r* values from 0.16 to 0.20, *p* < 0.05), and dizziness handicap (Spearman *r* values from 0.15 to 0.26, *p* < 0.01).

On the Motion Sickness Susceptibility Questionnaire, the participants with vestibular disease had lower scores than the participants without vestibular disease (Mann Whitney U test, adjusted *Z* = 2.041, *p* = 0.04). This score was related to the scores on: deviation of orientation on the object perspective test (Spearman *r* = −0.25, *p* < 0.00001), perceived stress (Spearman *r* = 0.24, *p* < 0.00001), and anxiety/depression (HADS) (Spearman *r* values from 0.17 to 0.34, *p* < 0.005).

**Figure 3:**
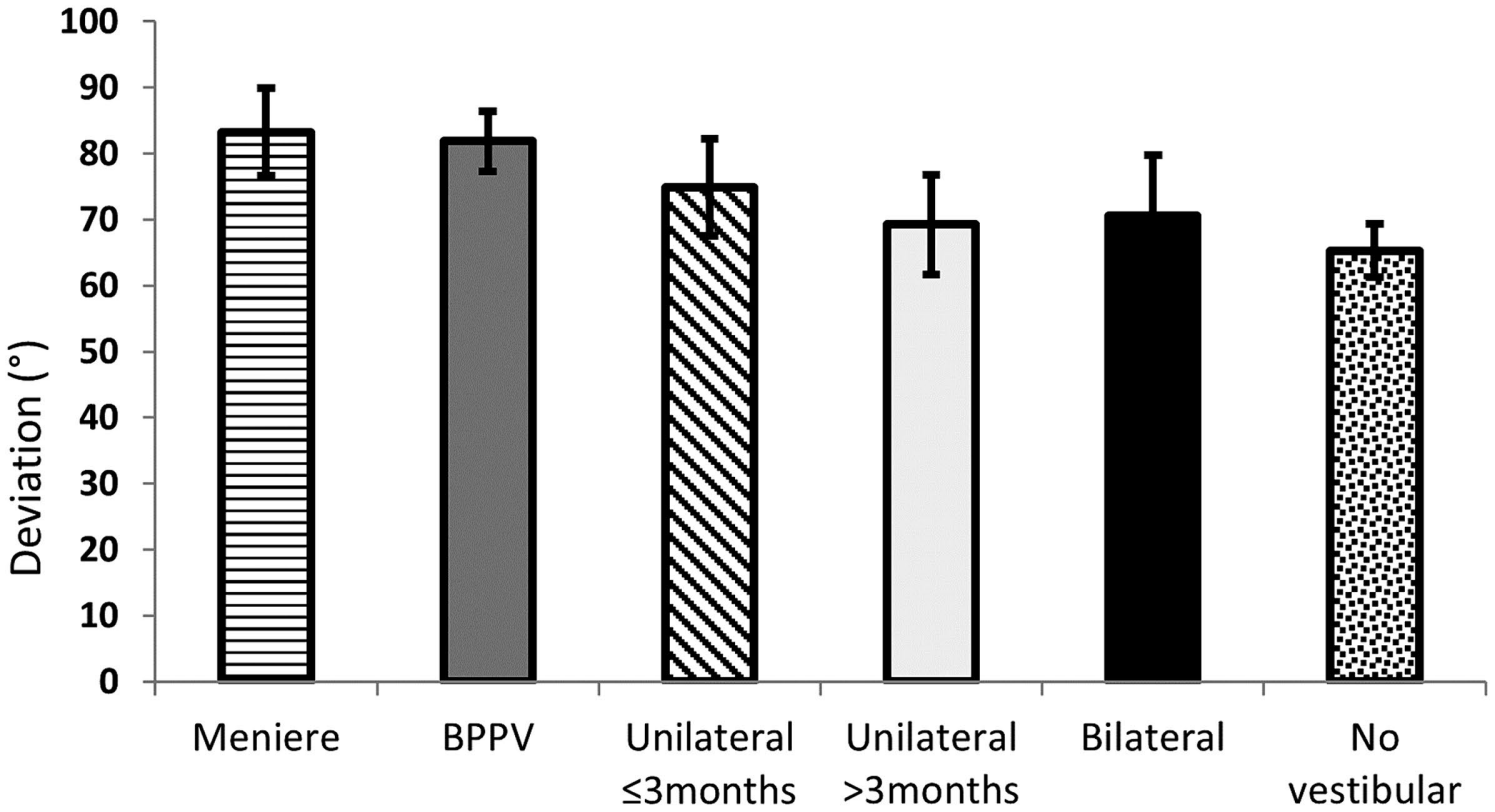
Mean and 95% Confidence Interval of the mean of the deviation on the Object Perspective Test of 153 patients (by general diagnosis) and 156 participants with no vestibular disease. (BPPV, Benign Paroxysmal Positional Vertigo).

On the Object Perspective Test, the participants without vestibular disease showed less total orientation deviation than the participants with vestibular disease (“*t*” test, *t* = 4.364, *p* = 0.0001). This difference was related to larger deviations in patients with either Meniere’s disease, and those with BPPV compared to participants without vestibular disease (ANOVA, *F* = 6.130, *p* < 0.0001; Tukey HSD test for unequal N, *p* < 0.05) (Figure 3). Among the participants with vestibular disease, patients with unilateral deficiency ≥3 months showed less deviation than those with BPPV (ANOVA, *F* = 3.872, *p* = 0.005; Tukey HSD test for unequal N, *p* = 0.01), with a borderline result when compared to patients with Meniere’s disease (Tukey HSD test for unequal N, *p* = 0.057). The deviation of orientation was related to the scores on: motion sickness susceptibility (Spearman *r* values from −0.25 to −0.22, *p* < 0.00002), dizziness handicap (Spearman *r* values from 0.19 to 0.23, *p* < 0.0005), state anxiety (Spearman *r* = 0.20, *p* < 0.0005), perceived stress (Spearman *r* = −0.11, *p* < 0.05), and spatial anxiety total score and the three sub-scores (Spearman *r* values from 0.11 to 0.15, *p* < 0.02).

**Figure 4:**
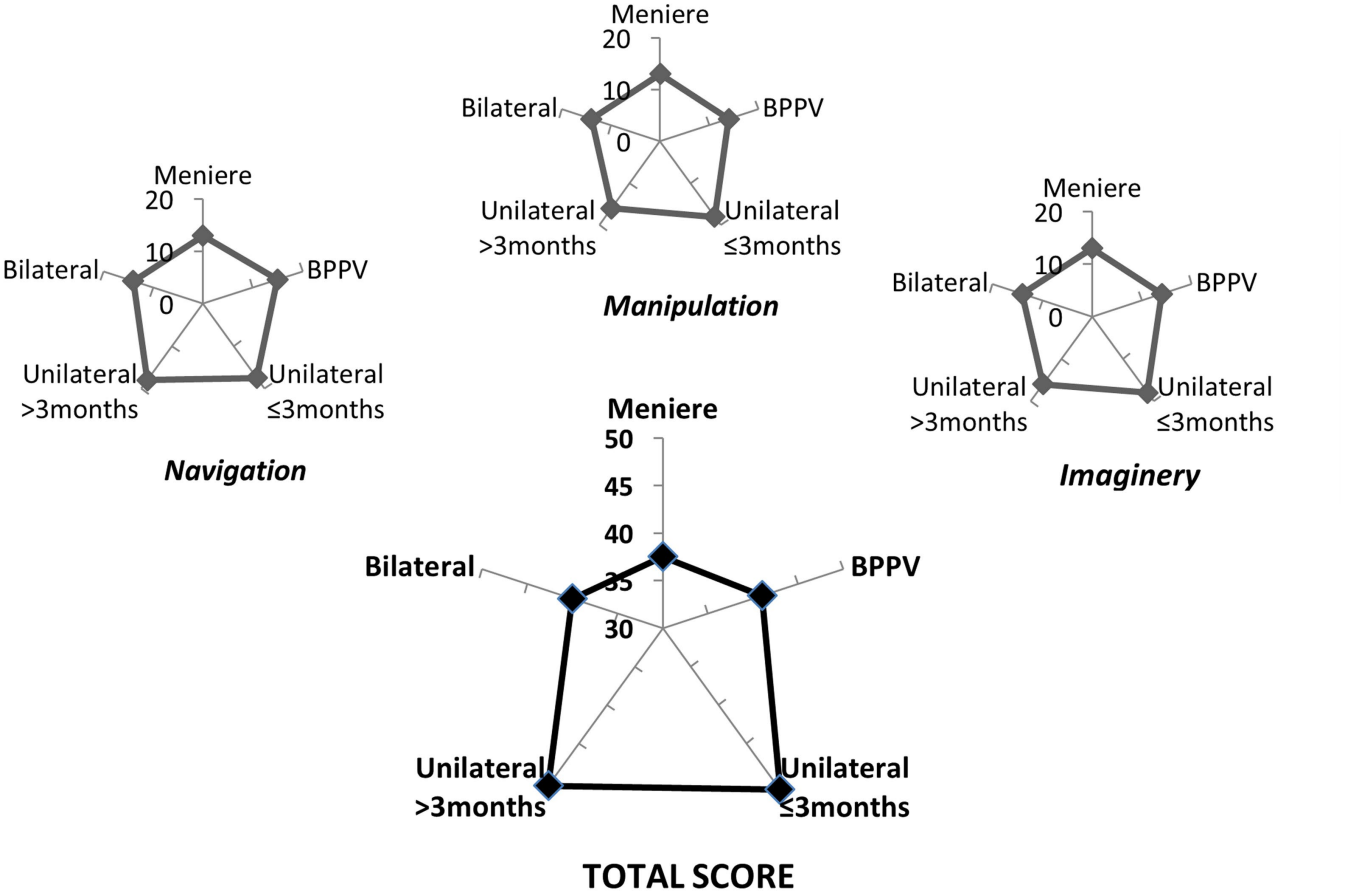
Median of the sub-scores and total score on the spatial anxiety of 153 patients by general diagnosis. (BPPV, Benign Paroxysmal Positional Vertigo).

On the Spatial Anxiety Scale, the three sub-scores and the total score were consistently higher in participants with vestibular disease compared to participants without vestibular disease (Table 3) (Mann Whitney U test, adjusted *Z* > 8.390, *p* < 0.00001). However, no significant difference was evident among the subgroups of participants with vestibular disease (*p* > 0.05) (Figure 4). Correlation between the scores (partial and total) on the Spatial Anxiety Scale and each of the other instrument scores is described in Table 4. Consistent correlations across the sub-scores and total score on spatial anxiety were observed on the scores on: symptoms related to unsteadiness (Spearman *r* values from 0.45 to 0.50; *p* < 0.00001), state anxiety (Spearman *r* values from 0.29 to 0.33, *p* < 0.00001), perceived stress (Spearman *r* values from 0.20 to 0.29, *p* > 0.0005), and the deviation of orientation on the object perspective test (Spearman *r* values from 0.11 to 0.15, *p* < 0.05).

**Table 4:** Spearman rank correlation between the Spatial Anxiety Questionnaire sub-scores and total score, and the scores on the instruments to evaluate the study variables, of 309 participants (153 with and 156 without peripheral vestibular disorder).

Domain Instruments	Navigation	Mental Manipulation	Imaginery	Total score
Questionnaire of symptoms	0.50**	0.48**	0.45**	0.50**
Anxiety sub-score	ns	ns	ns	ns
Depression sub-score	0.14*	ns	ns	ns
Total anxiety/depression score	0.12*	ns	ns	ns
State-Trait Anxiety (short version)	0.32**	0.29**	0.33**	0.33**
Perceived Stress	0.29**	0.20**	0.25**	0.26**
Depersonalization/derealization	0.20**	0.16**	0.11*	0.16**
Motion sickness total score	ns	ns	−0.12*	ns
Object Perspective Test	0.11*	0.15*	0.13*	0.13*

Coefficients are marked with asterisks according to statistical significance (ns, not significant, *p < 0.05 and **p < 0.005).

On the Dizziness Handicap Inventory, among the subgroups of participants with vestibular disease, a difference was observed on the emotional domain, in which patients with unilateral deficiency <3 months showed the highest score among all subgroups (Kruskal Wallis, *H* = 11.115, *p* = 0.02) (Table 3). Correlation between the scores (partial and total) on the handicap related to dizziness and each of the other instrument scores is described in Table 5. Consistent correlations of the sub-scores and total score on handicap related to dizziness were observed with the scores on: symptoms related to unsteadiness (Spearman *r* values from 0.66 to 0.79; *p* < 0.00001), spatial anxiety (Spearman *r* values from 0.47 to 0.50, *p* < 0.0002), state anxiety (Spearman *r* values from 0.36 to 0.38, *p* < 0.00001), anxiety/depression (HADS) (Spearman *r* values from 0.14 to 0.30, *p* < 0.05), depersonalization/derealization (Spearman *r* values from 0.15 to 0.27, *p* < 0.005), and deviation of orientation on the object perspective test (Spearman *r* from 0.19 to 0.22, *p* < 0.005).

**Table 5:** Spearman rank correlation between the Dizziness Handicap Inventory (sub-scores and total score) and the evaluation instruments of 309 participants (153 with and 156 without peripheral vestibular disease).

Domain Instruments	Physical	Emotional	Functional	Total
Questionnaire of symptoms	0.79**	0.66**	0.75**	0.78**
Anxiety HADS sub-score	ns	0.15*	0.15*	0.28**
Depression HADS sub-score	0.18**	0.26**	0.24**	0.27**
Total HADS score	0.14*	0.21**	0.21**	0.30**
State–Trait Anxiety (short version)	0.36**	0.37**	0.38**	0.38**
Perceived Stress	ns	ns	ns	ns
Depersonalization/derealization	0.23**	0.15*	0.27**	0.48**
Motion sickness Total score	ns	ns	ns	ns
Spatial anxiety Navigation	0.47**	0.34**	0.39**	0.43**
Spatial anxiety Mental Manipulation	0.50**	0.37**	0.42**	0.46**
Spatial anxiety Imagery	0.49**	0.36**	0.40**	0.44**
Spatial Anxiety Total	0.51**	0.37**	0.42**	0.46**
Object Perspective Test	0.22**	0.19**	0.22**	0.23**

Coefficients are marked with asterisks according to statistical significance (*p < 0.05 and **p < 0.005). HADS, Hospital Anxiety and Depression Scale.

### 3.2 Covariance analysis including cofactors

#### 3.2.1 Analysis on spatial anxiety score

**Figure 5:**
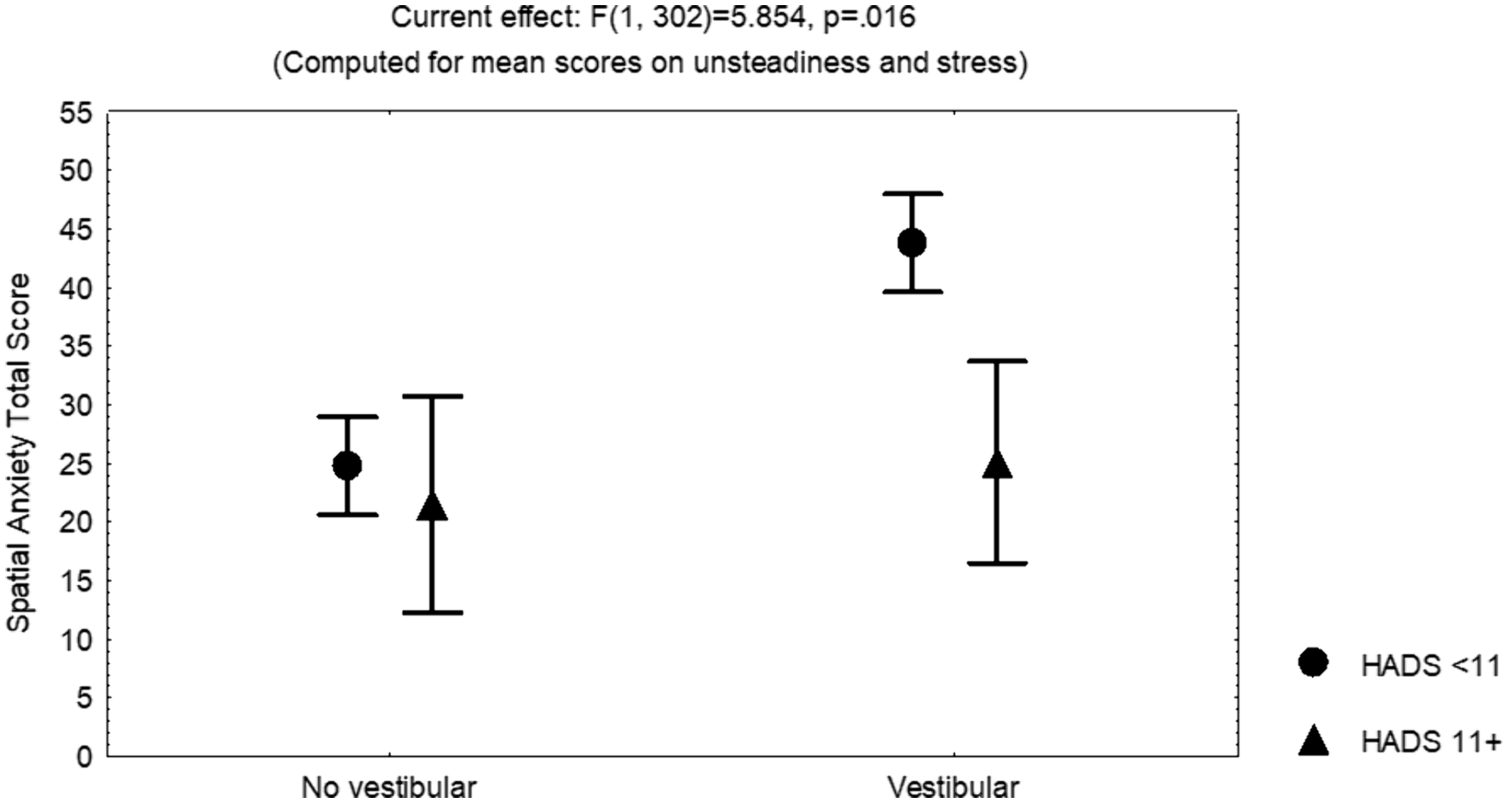
Least square means and standard error of the mean of the total score on the Spatial Anxiety Scale, corrected by the mean scores on symptoms of unsteadiness and perceived stress, of 156 participants without and 153 participants with vestibular disease. (HADS, Hospital Anxiety and Depression Scale).

The covariance analysis including individual cofactors showed that the variables consistently contributing to the variance across the total score and sub-scores on spatial anxiety were (ANCoVA, adjusted multiple *R*^2^ from 0.27 to 0.30, *F* values from 17.945 to 20.086, *p* < 0.00001) (beta values by domain are described in Table 6): symptoms related to unsteadiness, perceived stress, and HADS score ≥11 points, besides the contrast between participants with bilateral vestibular deficiency versus those without vestibular disease. Participants with vestibular disease with a HADS total score ≥11 points showed lower spatial anxiety sub-scores and total score than those with a HADS total score <11 points, after controlling for covariates (ANCoVA, *F* = 5.854, *p* = 0.016) (Figure 5). Further exploratory analysis showed that this difference was evident in all the subgroups of patients and the result was reproducible on the HADS anxiety sub-score (≥8) (ANCoVA, *F* = 5.282, *p* = 0.02), but not on the depression sub-score (≥8) (*p* > 0.05).

**Table 6:** Beta values and 95% Confidence Interval (C.I.) of the beta values of the variables included in the general linear model on the Spatial Anxiety score and subscores.

Domain	Navigation		Mental manipulation		Imagery		Total	
Factors	Beta (ß)	95% C.I.	Beta (ß)	95% C.I.	Beta (ß)	95% C.I.	Beta (ß)	95% C.I.
Questionnaire of symptoms	0.27	0.12–0.42	0.18	0.03–0.33	0.05	−0.10–0.20	0.18	0.03–0.32
Perceived Stress	0.28	0.17–0.38	0.21	0.11–0.32	0.26	0.15–0.36	0.26	0.15–0.36
HADS ≥8points	0.19	0.06–0.32	0.25	0.12–0.38	0.24	0.10–0.37	0.23	0.10–0.36
Bilateral deficiency versus:								
No vestibular disease	−0.18	−0.34--0.02	−0.30	−0.45–0.14	−0.38	−0.54–0.22	−0.30	−0.45–0.14
Meniere’s disease	−0.05	−0.18–0.08	0.04	−0.10–0.17	0.01	−0.12–0.15	0.00	−0.13–0.13
Benign Paroxysmal Positional Vertigo	0.00	−0.13–0.14	0.00	−0.13–0.13	0.05	−0.09–0.18	0.02	−0.11–0.15
Unilateral <3 months	0.00	−0.16–0.15	0.05	−0.10–0.20	0.02	−0.13–0.18	0.02	−0.13–0.18
Unilateral >3 months	0.08	−0.05–0.20	0.04	−0.08–0.16	0.06	−0.06–0.18	0.06	−0.06–0.18
HADS ≥8points * Bilateral deficiency versus:								
No vestibular disease	−0.13	−0.27–0.02	−0.14	−0.28–0.00	−0.15	−0.29–0.01	−0.15	−0.29–0.01
Meniere’s disease	0.03	−0.10–0.16	0.01	−0.12–0.14	0.07	−0.06–0.20	0.04	−0.09–0.17
Benign Paroxysmal Positional Vertigo	0.07	−0.06–0.20	0.06	−0.07–0.20	0.06	−0.07–0.20	0.07	−0.06–0.20
Unilateral <3 months	0.05	−0.11–0.20	0.03	−0.12–0.18	0.00	−0.15–0.16	0.03	−0.12–0.18
Unilateral >3 months	0.04	−0.08–0.16	0.09	−0.03–0.21	0.08	−0.04–0.20	0.07	−0.05–0.19

HADS, Hospital Anxiety and Depression Scale.

#### 3.2.2 Analysis on the orientation deviation score

**Figure 6:**
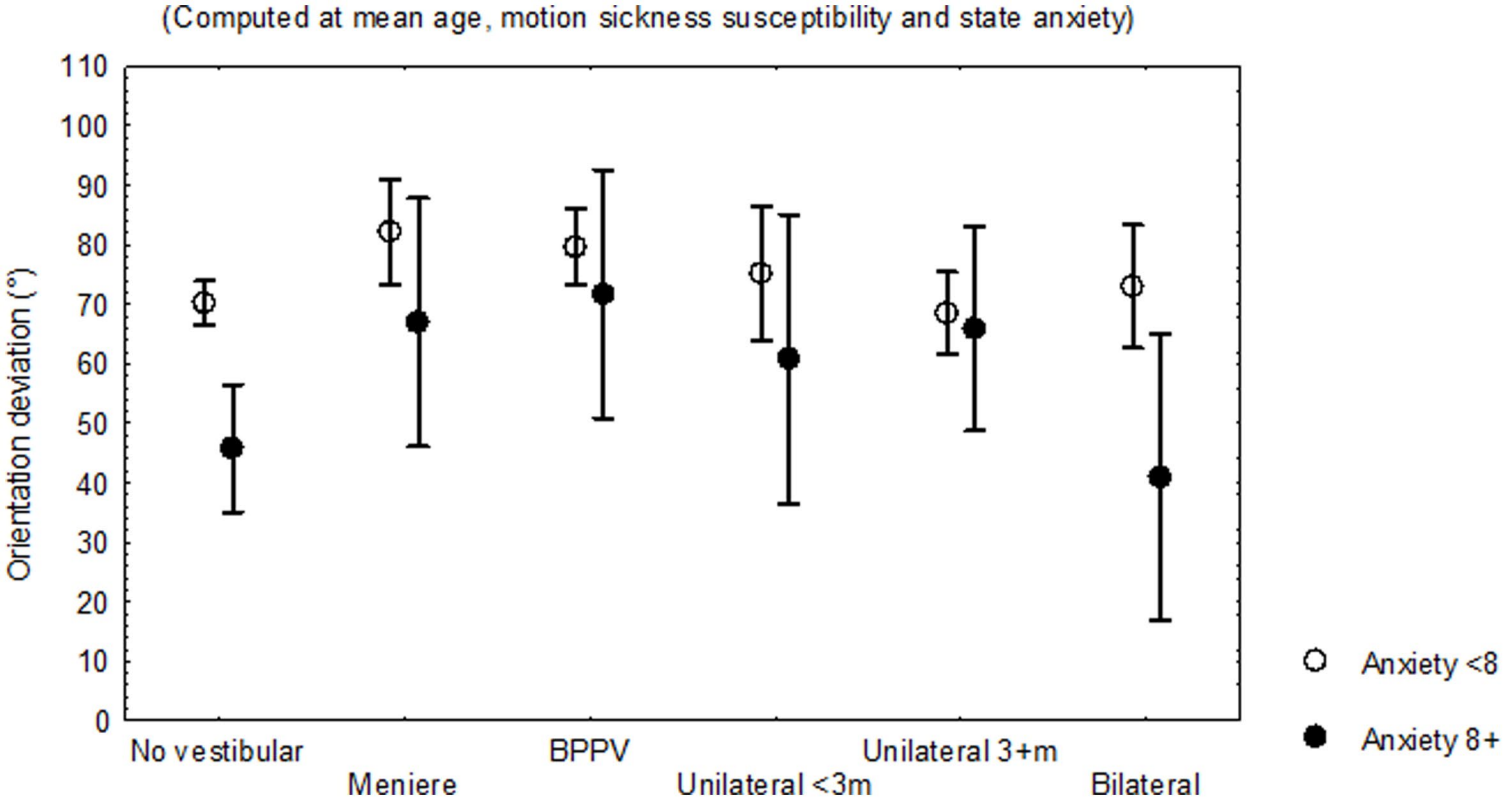
Least square means and standard error of the mean of the deviation of orientation on the Objective Perspective Test, according to the evidence of trait anxiety, corrected by the mean age, motion sickness susceptibility score and state anxiety score of 156 participants without and 153 participants with vestibular disease, by subgroup of diagnoses.

The covariance analysis including individual cofactors showed that the variables consistently contributing to the variance on the orientation deviation were (ANCoVA, adjusted multiple *R*^2^ = 0.18, *F* = 5.834, *p* < 0.00001): vestibular disease (beta −0.23, 95% C.I. −0.40–0.05), HADS anxiety sub-score (≥8) (beta 0.22, 95% C.I. 0.09–0.34) (Figure 6), state anxiety (beta 0.20, 95% C.I. 0.08–0.31), motion sickness susceptibility (beta −0.15, 95% C.I. −0.25–0.04), and age (beta 0.11, 95% C.I. 0.001–0.21); however no interaction between the vestibular diagnose and HADS anxiety sub-score (≥8) was observed.

#### 3.2.3 Analysis on the dizziness handicap inventory score and subscores

The covariance analysis including individual cofactors showed that the variables consistently contributing to the variance on the total Dizziness Handicap Inventory score were (ANCoVA, adjusted multiple *R*^2^ = 0.66, *F* = 39.07, *p* < 0.00001) (beta values are described in Table 7): symptoms related to unsteadiness; orientation deviation; quality of sleep score; HADS score ≥11 points, with higher handicap score in those with HADS score ≥11 points and either unilateral deficiency <3 months or BPPV; and the contrast between participants with bilateral vestibular deficiency versus those without vestibular disease, those with unilateral deficiency <3 months, and those with BPPV.

**Table 7:** Beta values and 95% Confidence Interval (C.I.) of the beta values of the variables included in the general linear model on the Dizziness Handicap Inventory score and sub-scores.

Domain	Physical		Emotional		Functional		Total	
Factors	Beta (ß)	95% C.I.	Beta (ß)	95% C.I.	Beta (ß)	95% C.I.	Beta (ß)	95% C.I.
Questionnaire of symptoms	0.35	0.24–0.46	0.16	0.03–0.29	0.36	0.24–0.49	0.31	0.20–0.43
Spatial Anxiety total score	0.09	0.02–0.17	−0.06	−0.16–0.03	−0.09	−0.17–0.00	−0.02	−0.10–0.06
Orientation deviation (Object Perspective Test)	0.07	0.00–0.13	0.08	0.00–0.17	0.09	0.01–0.17	0.08	0.01–0.16
Sleep Quality Index score	0.07	−0.01–0.13	0.10	0.01–0.19	0.08	0.00–0.16	0.09	0.02–0.16
HADS ≥11points	−0.09	−0.16–0.01	−0.15	−0.25–0.05	−0.17	−0.26–0.08	−0.15	−0.23 –0.07
Bilateral deficiency versus:								
No vestibular disease	−0.55	−0.67–0.42	−0.69	−0.85–0.54	−0.58	−0.72–0.44	−0.65	−0.78–0.52
Meniere’s disease	−0.05	−0.16–0.05	−0.08	−0.21–0.05	−0.09	−0.21–0.03	−0.08	−0.19–0.03
Benign Paroxysmal Positional Vertigo	0.20	0.09–0.32	0.18	0.03–0.32	0.18	0.05–0.31	0.20	0.08–0.32
Unilateral <3 months	0.27	0.16–0.37	0.36	0.23–0.50	0.38	0.26–0.51	0.36	0.25–0.48
Unilateral >3 months	0.04	−0.05–0.11	−0.06	−0.18–0.07	0.09	−0.02–0.20	0.03	−0.07–0.13
HADS ≥11 points * Bilateral deficiency versus:								
No vestibular disease	0.15	0.04–0.25	0.15	0.02–0.29	0.20	0.08–0.33	0.18	0.07–0.29
Meniere’s disease	0.09	0.01–0.20	0.03	−0.10–0.16	0.04	−0.08–0.16	0.06	−0.05–0.17
Benign Paroxysmal Positional Vertigo	−0.12	−0.23–0.01	−0.30	−0.44–0.16	−0.21	−0.33–0.08	−0.23	−0.34–0.11
Unilateral <3 months	−0.18	−0.29–0.07	−0.14	−0.28–0.00	−0.23	−0.36–0.11	−0.20	−0.31–0.09
Unilateral >3 months	−0.01	−0.11–0.09	0.12	0.00–0.25	0.00	−0.11–0.11	0.04	−0.06–0.14

HADS, Hospital Anxiety and Depression Scale.

The covariance analysis on each domain of the Dizziness Handicap Inventory showed that the variables that consistently contributed to the variance on the Physical domain score were (ANCoVA, adjusted multiple *R*^2^ = 0.69, *F* = 44.77, *p* < 0.00001) (Table 7): symptoms related to unsteadiness, the orientation deviation, spatial anxiety (total score and sub-scores), quality of sleep, HADS score ≥11 points, and the contrast between participants with bilateral vestibular deficiency versus those without vestibular disease, those with unilateral deficiency <3 months, and those with BPPV. The variables that consistently contributed to the variance on the Emotional domain were (ANCoVA, adjusted multiple *R*^2^ = 0.52, *F* = 21.32, *p* < 0.00001) (Table 7): symptoms related to unsteadiness, quality of sleep, HADS score ≥11 points, and the contrast between participants with bilateral vestibular deficiency versus those without vestibular disease, and those with unilateral deficiency <3 months. The variables that consistently contributed to the variance on the Functional domain were (ANCoVA, adjusted multiple *R*^2^ = 0.60, *F* = 29.53, *p* < 0.00001) (Table 7): symptoms related to unsteadiness, orientation deviation, quality of sleep score, HADS score ≥11 points, and the contrast between participants with bilateral vestibular deficiency versus those without vestibular disease, and those with unilateral deficiency <3 months; however when the interaction between HADS ≥11 points and the vestibular diagnoses was considered, a significant result was also observed for the subgroup with BPPV. Additionally, the Navigation domain of the spatial anxiety questionnaire also contributed to the variance on the Functional domain of dizziness-related handicap (ANCoVA, *F* = 4.940, *p* = 0.027).

## 4 Discussion

In this study, spatial anxiety was related to the report of both symptoms of unsteadiness and perceived stress, but it was less in those with HADS anxiety sub-score ≥ 8 (Figure 5). The total score and the sub-scores on spatial anxiety contributed to the variability on the score of the Physical domain of the Dizziness Handicap Inventory, while just the score of the Navigation domain contributed to the functional domain of the handicap instrument. Other variables contributing to the variability on dizziness-related handicap were the report of symptoms of unsteadiness, the orientation deviation on the object perspective test, the quality of sleep, and anxiety/depression symptoms. Additionally, the orientation deviation was related to vestibular disease, the score on anxiety (both state anxiety and trait anxiety), the motion sickness susceptibility, and age (Figure 6).

The simultaneous relationship of spatial anxiety with symptoms of unsteadiness and perceived stress could be considered within the response to vestibular damage. Stress is a physiological reaction to a stimulus, improving the opportunity to overcome a stressor, which varies according to the stressor and the individual characteristics. Image studies support that down-regulation of the fear network may contribute to reduce distress during unpleasant body accelerations (63); while in patients with acute vestibular damage, acute stress facilitates vestibular compensation (33). However, repetitive exposure to stressors may induce adaptive changes in the brain circuits regulating the stress response (64), while removing the emotional arousal of novelty may abolish facilitation (65).

Chronic stress provokes circumscribed neurochemical changes in sub-regions of the hippocampus and the entorhinal and frontal cortices (66). In animal models, chronic stress is a cause of neuron atrophy in both the hippocampus and the prefrontal cortex, which are involved in memory, selective attention, and executive function, with hypertrophy of neurons in the amygdala that is involved in fear and anxiety, as well as aggression (67), and it also disturbs neurogenesis and survival of newly born neurons in the hippocampus (68); consequently, chronic stress alters the acute stress response on functions such as spatial memory (69). Consistently, patients with chronic bilateral vestibular deficits may report spatial anxiety (12), and display deficits on spatial abilities (12, 70, 71).

In this study, participants with peripheral vestibular disease and HADS anxiety sub-score ≥ 8 (symptoms of trait anxiety) showed lower spatial anxiety, compared to those with HADS anxiety sub-score < 8 (less symptoms of trait anxiety) (Figure 5), while participants with trait anxiety showed less orientation deviation on the object perspective test. These findings are consistent with studies in healthy adults within a threatening context, in which high levels of trait anxiety may improve the ability to retrace a route, whereas low levels of trait anxiety may be associated with worse performance under threat, supporting that the predisposition for emotional reaction could be helpful to overcome the apprehension provoked by the spatial task (26). Additionally, the interrelated neural circuits controlling stress and anxiety substantiate a bidirectional relationship (34); in subjects without psychiatric diagnosis, anxiety and depression have been associated with blunted or exaggerated cortisol responses to and recovery from stress (72).

It is frequently assumed that vestibular symptoms are provoked/heightened by anxiety and stress, but the results of this study support that state anxiety and the stress response could be helpful for recovery, whereas spatial anxiety can be detrimental. The results support that, in patients with peripheral vestibular disease, spatial anxiety may contribute to dizziness-related handicap. This contribution was evident particularly on the Physical domain of the handicap instrument, besides the distinct contribution of the Navigation domain of spatial anxiety to the Functional domain of the handicap inventory. These results are congruent with the design of the instruments administered for evaluation of these two variables. The Spatial Anxiety Questionnaire refers “to situations and experiences that may cause tension, apprehension, or anxiety” (31). In this study, the participants with peripheral vestibular disease consistently reported spatial anxiety related to performance of physical activities, including activities that may provoke dizziness/unsteadiness, which are evaluated by the Physical domain of the Dizziness Handicap Inventory (60); while anxiety related to navigation was deleterious for their daily life functioning, according to the limiting consequences of the symptoms reported by the Functional domain of the handicap inventory. These concepts are also classified in the International Classification of Functioning, Disability and Health (73) and described as: “activity limitations are difficulties an individual may have in executing activities,” and “participation restrictions are problems an individual may experience in involvement in life situations.” Yet, variability of limitations and restrictions related to health conditions may be influenced by contextual factors, including personal factors such as “fitness, lifestyle, habits, upbringing, coping styles, social background, education, profession, past and current experience (past life events and concurrent events), overall behavior pattern and character style, individual psychological assets and other characteristics” (73).

The contribution of the report of unsteadiness and symptoms of anxiety/depression (HADS) to the dizziness-related handicap is consistent with evidence towards worse scores on self-reported measurements on functioning increasing the severity of dizziness-related handicap (74), and the correlation between the report of symptoms of depression with both vestibular symptoms and the sense of disability (75). Consistently, after acute vestibular lesion, depression symptoms may persist while updating spatial orientation improves (45); whereas symptomatic peripheral vestibular disease has been related to increased risk for attempted suicide over a follow-up of 1 year, after adjusting for demographic related comorbidities (76). Although, adaptive changes may occur that could explain the differences among the subgroups of patients with variable clinical evolution. Additionally, the report of depersonalization/derealization symptoms was linearly related to multiple variables, including unsteadiness, perceived stress, and anxiety/depression, yet the multivariate analysis did not show an independent contribution to the variability of dizziness-related handicap.

The contribution of the orientation deviation to handicap, particularly in patients with episodic vertigo, can be explained by the relevance of the vestibular reference for egocentric mental transformations (77); though perspective-taking tests can be solved by both mentally reorienting oneself and mentally rotating the stimuli (78). Additionally, the orientation deviation had a negative relationship with the score on motion sickness susceptibility, which was less in patients with bilateral vestibular deficit. This result is consistent with the requirement of intact vestibular function for the provocation of motion sickness and to the association of motion sickness with mechanisms involved in adapting the spatial orientation system to strange environments (79).

The contribution of the quality of sleep to dizziness-related handicap corresponds to the evidence on the interaction of sleep with vestibular function and balance (80–82). Sleep and wakefulness are regulated by the aminergic, cholinergic brainstem and hypothalamic systems with involvement of immune homeostatic regulating mechanisms (83). In rats, vestibular damage can affect the sleep-wakefulness cycle with up-regulation of the level of autophagy in hypothalamic tissue (84). In humans, epidemiologic evidence supports that adults with vertigo have a higher risk for abnormal sleep duration (85); conversely, in patients with sleep complains, sleep architecture variation has been associated with vestibular symptoms (86). Additionally, clinical studies have shown that persistent sleep disturbance after vestibular rehabilitation is related to handicap severity (87), while rehabilitation for unilateral vestibular disease is associated to improvement of the quality of sleep (88). However, these variables interact with stress, with a bidirectional relation between stress and sleep quantity, in which worse sleep quantity and continuity may predict higher next-day stress (89).

The findings of this study may support future studies, including the design of clinical studies on spatial anxiety and chronic stress in adults with peripheral vestibular disease, to assess their spatial abilities, according to their clinical characteristics. Of relevance to rehabilitation, it is possible that spatial anxiety, specifically apprehension about moving in situations which would provoke unpleasant, vestibular symptoms, could prevent a patient from actively engaging with challenging circumstances to encourage adaptation.

The results of this study should be interpreted in the context of its limitations. The cross-sectional design prevents discussion of any causal relationship. The sample size was calculated to assess moderate to strong correlations; thus, we cannot deny weaker relationships. The study was limited to the most obvious factors influencing the results. Enrolment was limited to patients receiving specialized medical care without comorbidities that could interfere with the assessed relationships, so the results may be different in other clinical settings. However, the strengths of the study include the prospective collection of the data by trained health professionals, the variety of cofactors that were evaluated, and the consistency of the results.

## 5 Conclusion

In adults both with and without vestibular disease, spatial anxiety can be related to both unsteadiness and perceived stress and may be less in those with symptoms of trait anxiety (HADS anxiety sub-score ≥ 8). Spatial anxiety and impairment of perspective-taking, along with poor quality of sleep and trait anxiety may contribute to the Physical and Functional domains of dizziness-related handicap. Whereas follow-up studies are required to assess if a degree of anxiety and stress could be beneficial to encourage rehabilitation, specific spatial anxiety can be detrimental, possibly because it limits behavior beneficial to adaptation.

## Data availability statement

The raw data supporting the conclusions of this article will be made available by the authors, without undue reservation.

## Ethics statement

The studies involving humans were approved by Instituto Mexicano del Seguro Social. IMSS R 2021-3601-219. The studies were conducted in accordance with the local legislation and institutional requirements. The participants provided their written informed consent to participate in this study.

## Author contributions

KJ-R: Conceptualization, Formal analysis, Investigation, Methodology, Project administration, Resources, Supervision, Visualization, Writing – original draft, Writing – review & editing. DG-J: Data curation, Investigation, Validation, Writing – review & editing. SB-O: Data curation, Investigation, Validation, Writing – review & editing. MG: Visualization, Writing – review & editing. AG-M: Investigation, Validation, Writing – review & editing.
